# Impact of universal medical insurance system on the accessibility of medical service supply and affordability of patients in China

**DOI:** 10.1371/journal.pone.0193273

**Published:** 2018-03-07

**Authors:** Xiaolei Xiong, Zhiguo Zhang, Jing Ren, Jie Zhang, Xiaoyun Pan, Liang Zhang, Shiwei Gong, Si Jin

**Affiliations:** 1 Department of Pharmacy Business and Administration, School of Pharmacy, Tongji Medical College of Huazhong University of Science and Technology, Wuhan, Hubei, China; 2 School of Medicine and Health Management, Tongji Medical College of Huazhong University of Science and Technology, Wuhan, Hubei, China; 3 New Rural Cooperative Medical Research Center of National Health and Family Planning Commission, Beijing, China; 4 Health Insurance Research Association of Ministry of Human Resources and Social Security, Beijing, China; 5 Department of Pharmaceutical Systems and Policy, School of Pharmacy, West Virginia University, Morgantown, West Virginia, United States of America; 6 Department of Endocrinology, Institute of Geriatric medicine, Liyuan Hospital, Tongji Medical College of Huazhong University of Science and Technology, Wuhan, Hubei, China; Dartmouth-Hitchcock Medical Center, UNITED STATES

## Abstract

**Background:**

China’s universal medical insurance system (UMIS) is designed to promote social fairness through improving access to medical services and reducing out-of-pocket (OOP) costs for all Chinese. However, it is still not known whether UMIS has a significant impact on the accessibility of medical service supply and the affordability, as well as the seeking-care choice, of patients in China.

**Methods:**

Segmented time-series regression analysis, as a powerful statistical method of interrupted time series design, was used to estimate the changes in the quantity and quality of medical service supply before and after the implementation of UMIS. The rates of catastrophic payments and seeking-care choices for UMIS beneficiaries were selected to measure the affordability and medical service flow of patients after the implementation of UMIS.

**Results:**

China’s UMIS was established in 2008. After that, the trending increase of the expenditure of the UMIS was higher than that of increase in revenue compared to previous years. Up to 2014, the UMIS had covered 97.5% of the entire population in China. After introduction of the UMIS, there were significant increases in licensed physicians, nurses, and hospital beds per 1000 individuals. In addition, hospital outpatient visits and inpatient visits per year increased compared to the pre-UMIS period. The average fatality rate of inpatients in the overall hospital and general hospital and the average fatality rate due to acute myocardial infarction (AMI) in general hospitals was significantly decreased. In contrast, no significant and prospective changes were observed in rural physicians per 1000 individuals, inpatient visits and inpatient fatality rate in the community centers and township hospitals compared to the pre-UMIS period. After 2008, the rates of catastrophic payments for UMIS inpatients at different income levels were declining at three levels of hospitals. Whichever income level, the rate of catastrophic payments for inpatients of Urban Employee’s Basic Medical Insurance was the lowest. For the low-income patients, a single hospitalization at a tertiary hospital can lead to catastrophic payments. It is needless to say what the economic burden could be if patients required multiple hospitalizations within a year. UMIS beneficiaries showed the intention of growth to seek hospitalization services in tertiary hospitals.

**Conclusions:**

Introduction of the UMIS contributed to an increase in available medical services and the use thereof, and a decrease in fatality rate. The affordability of UMIS beneficiaries for medical expenses was successfully ameliorated. The differences in patients’ affordability are mainly manifested in different medical insurance schemes and different seeking-care choices. The ability of the poor patients covered by UMIS to resist catastrophic medical payments is still relatively weak. Therefore, the current UMIS should reform the insurance payment model to promote the integration of medical services and the formation of a tiered treatment system. UMIS also should establish supplementary medical insurance packages for the poor.

## Introduction

Medical insurance is a means of protection from financial risk of incurring medical expenses. A medical insurance system is generally designed to improve accessibility and equity of health service utilization and to protect their population from medical impoverishment [1]. China is a developing country with the largest population worldwide. The Chinese government is facing a challenge to ensure accessibility and affordability of universal health care services. Since the foundation of the People’s Republic of China in 1949, the Chinese government has been committed to setting up a universal medical insurance. As early as 1951 and 1952, the Chinese government had set up a free health care service system of labor insurance schemes (LIS) and government employee insurance scheme (GIS), respectively. LIS was an enterprise unit-based self-insurance system that covered partial medical costs for workers. Lineal dependents were often included in this system [2]. In 1959, the Ministry of Health of China affirmed the form of rural cooperative medical scheme (RCMS). Up to 1976, in over 90 percent of administrative villages in China, RCMS was established. At that point in time, most Chinese were covered by the GIS, LIS or RCMS under the condition of meager medical resources [3]. However, starting from 1978, China began to implement a new social economic transformation according to the requirement of the Third Plenary Session of the Eleventh Central Committee of the Communist Party of China. Since then, China’s economic system changed from a planned economy to a market economy. Due to the ownership reform of state-owned units, the rapid growth of medical costs and massive waste of excessive consumption, the funds of GIS and LIS displayed gradually deficit. A study by Tianxiao et al. showed that the number of beneficiaries of GIS and LIS reached 100 million in 1988 as compared to 73 million in 1978. The growth was 37% and total expenses increased 314% [4]. Since 1980, the growth rate of health expenditure was 18.9% beyond 10% growth rate of China’s fiscal revenue. Therefore, the central government had to reform the free health service system. With the implementation of a household contract responsibility system, the collective economic model altered in rural areas, which resulted in a nearly collapse of the RCMS. In 1985, the RCMS in only 5 percent of administrative villages in China was still intact [3].

In 1978, the government health expenditures dropped from 32.2% of total health expenditures to 16.4% in 1997. In addition, the economic reform unexpectedly weakened the function of GIS, LIS, and RCMS [5]. At the end of 1998, China launched a mandatory insurance program, the urban employee basic medical insurance (UEBMI), to replace the previous GIS and LIS system. This system was modeled on a social medical insurance in Germany and a savings medical insurance in Singapore. The UEBMI expanded coverage to private sector employees and provided a mutual sharing risk pool of insurance costs by employers and individual workers at the city level [2]. Up to 2011, the GIS was cancelled in 24 out of 31 provinces [6]. In 2003, the new rural cooperative medical scheme (NRCMS) was re-established to reduce the risk of catastrophic health spending for residents in rural areas. Between 1982 and 2003, China’s rural residents were void of the formal medical insurance system. In 2003, the Third National Health Service Survey (NHSS) of China showed that 44.8% of the urban population and 79.1% of the rural population did not have medical insurance coverage. Of all the surveyed households, 30% self-reported that their poverty situation was primarily due to illness or injuries [7]. Therefore, disease had become one of the key causes of poverty in China. The NRCMS is a government-run voluntary insurance program that is jointly financed by central and local governments and individual households. In 2007, the urban residents’ basic medical insurance (URBMI) was established for urban residents, including the unemployed, elderly people, children and students. Thus, the policy system of universal medical insurance was reformed in 2007. The medical insurance system creates a link between hospitals and patients. The expansion of medical insurance coverage will stimulate more people to get medical care. Payments and co-payments associated with the medical insurance system will affect the quantities and ways of the hospitals’ and physicians’ services and quantities, cost and seeking-care flow of medical consumption of patients. It had been reported that, in 2000, the total amount that was paid by medical insurance funding accounted for roughly 3% of the total business income of medical institutions. In 2014, this percentage was 52.1% [8]. This suggests that medical insurance and hospital development are closely related.

A study by Xu et al. used data from the 1998 and 2003 NHSS of China to examine the impact of the 1997 urban medical insurance reform on population coverage. It was found that the proportion of the urban population without any medical insurance arrangement was similar between 1998 and 2003 [9]. In addition, a study by Meng et al., in which the data from the 2003, 2008, and 2011 NHSS of China was used, demonstrated that enormous strides had been made in achieving equal access to services and insurance coverage. However, the incidence of catastrophic health expenses for households did not decline between 2003 and 2011 [10]. A report by Pan et al. report showed that, in 2011, 13.8% of rural and 10.9% of urban households experienced catastrophic health expenditure [11]. Liang et al. conducted a systematic review of the effect of NRCMS and concluded it was not clear if NRCMS improved health outcomes and attenuated catastrophic health expenditure of the China’s rural population [12]. Zhang et al. collected data on the medical spending of rural households covered by NRCMS from 25 counties in China, which suggested that NRCMS had an increasing but small effect on the reduction of catastrophic medical payment incidence [13]. Chen et al. used data of the URBMI Survey (2008–2011) and found that the URBMI significantly increased the likelihood of receiving inpatient treatment in the past year. However, the insurance effect on reducing the underutilized hospitalization was insignificant [14]. Zhou et al. used the cross-sectional data from the 4th NHSS of Shaanxi province, and found that basic medical insurance schemes demonstrated a positive but limited effect on increasing health services utilization in the Shaanxi province [15]. In 2012, an editorial of the Lancet pointed out China’s health services were described as too difficult to access, too expensive, and too variable in quality [16].

These studies reveal that there is no longer a scarcity of medical material resources in today’s China. The fact that patient access is inadequate and medical care costs are high remains a major problem. It is important to know whether the UMIS improves the accessibility and affordability of medical services. Our study for the first time focuses on analyzing the changes of the quantity and quality of medical care supply at the population level and hospital level, as well as the economic burden and seeking-care flow for patients under the UMIS, trying to address the relationship between these changes and the current main problem.

## Methods

### Evaluation indicators

Adequate licensed physicians, nurses, and hospital beds are prerequisites for patients to access medical services. In our study, accessibility of medical services is defined as the extent to which the quantity and quality of medical service supplies are available. The quantity of medical services includes the number of practicing physicians, nurses, hospital beds as well as outpatient visits and inpatient visits referring to the European Core Health Indicators (ECHI) of the European Commission (EC) [17]. To evaluate the quantity of hospital care, the number of outpatient and inpatient visits per hospital was calculated. The number of visits per hospital equals the total number of hospital visits per year divided by the total number of hospitals available.

To assess the quality of medical services, we focused on evaluating hospital care quality [18]. In the United States (US), the Agency for Healthcare Research and Quality (AHRQ) used Inpatient Quality Indicators (IQIs) to assess the quality of hospital care. IQIs include inpatient mortality for several procedures and medical conditions (such as AMI, heart failure, acute stroke, hip fracture, gastrointestinal hemorrhage and pneumonia) as well as utilization and volume indicators for procedures [19]. In addition, the Centres for Medicaid and Medicare Services (CMS) adopted care quality measures of Hospital Quality Alliance (HQA) for US hospitals, including hospital 30-day mortality rates for heart attack, heart failure, and pneumonia [20]. In Europe, EC set up an indicator concerning quality of health care of 30-day in-hospital case-fatality of AMI and ischemic stroke in the ECHI indicator system [21].

In addition, the latest study of Healthcare Access and Quality Collaborators of the Global Burden of Diseases, Injuries, and Risk Factors Study (GBD) showed that China was one of countries with the largest increase in the Healthcare Quality and Access (HAQ) Index level in 2015. The average HAQ index of China was 74, compared with 53.7 for the average global HAQ Index in 2015. However, leukemia, rheumatic heart disease, cerebrovascular disease, and tuberculosis had a much higher contribution to the population mortality rate in China than the average level in Japan, Korea, Germany, the United Kingdom and the US [22]. Therefore, combined with the availability of data in China, we selected the average fatality rate of inpatients and average fatality rates of AMI, heart failure, pneumonia, leukemia, rheumatic heart disease, cerebrovascular disease, diabetes mellitus, and tuberculosis after hospital admission as quality evaluation indicators of medical services in China. In China, general hospitals are the representative body of high-tech medical services. The annual fatality rate of the eight diseases mentioned above in general hospitals were selected.

For the patients, the rate of catastrophic payment was selected to evaluate the affordability of medical services. The flow of medical services was assessed by the rate of seeking-care choice of inpatients for different-level hospitals.

### Data sources

The data of all evaluation indicators were obtained from the following sources.

1. Annual revenue and expenditure amount of UEBMI and URBMI between 2002 and 2014, extracted from the China Statistical Yearbook 2003 to 2015 [23]. Annual revenue and expenditure amount of NRCMS between 2002 and 2014 extracted from the China Health Statistics Yearbook 2003 to 2015. Annual population covered by the UEBMI, URBMI, and NRCMS between 2002 and 2014, extracted from the China Health Statistics Yearbook 2003 to 2015 [24, 25].
2. Annual numbers of different hospitals, licensed physicians and assistant physicians, nurses, hospital beds, outpatient and inpatient visits and case fatality rate of hospital between 2002 and 2014, all extracted from the China Health Statistics Yearbook 2003 to 2015 [24, 25].
3. Annual average medical expenses, out of pocket expenses, reimbursement rate and seeking-care choice of inpatients covered by UEBMI and URBMI from 2008 to 2014, obtained from the national sample survey on medical service utilization of basic medical insurance participants of the China Health Insurance Research Association from 2009 to 2015.
4. Annual average medical expenses, out of pocket expenses, reimbursement rate and seeking-care choice of inpatients covered by NRCMS from 2009 to 2015 were obtained from a database of the New Rural Cooperative Medical Research Center of China.
5. Three-levels of disposable incomes of residents were obtained between 2008 and 2014 from the China Statistical Yearbook 2009 to 2015 [23].

### Statistical description

Interrupted time series analysis is a quasi-experimental method for evaluating longitudinal effects of policy interventions [26]. The Segmented Time-Series Regression (STSR) model estimates the level and trend of the dependent variable before the implementation of UMIS and the changes in level and trend after the implementation of UMIS. The linear regression model was as follows [27]:

$${\text{Y}}_{t}=\beta_{0}+\beta_{1}*{\text{time}}_{t}+\beta_{2}*{\text{intervention}}_{t}+\beta_{3}*\text{time}\;\text{after}\;{\text{intervention}}_{t}+{\text{e}}_{t}$$

Y_*t*_ represents the mean value of each evaluation indicator of medical service supply in year t; time represents a continuous variable indicating time in years at time *t* from the start of the observation period; intervention represents an indicator for time t occurring before (intervention = 0) or after (intervention = 1) implementation of the UMIS. The start time of implementation of the UMIS was defined in 2008, and time after intervention represents a continuous variable counting the number of years after the intervention at time t, coded 0 before the UMIS (from 2002 to 2007) and coded 1–7 after the UMIS (from 2008 to 2014).

In this model, β_0_ estimates the baseline level of each evaluation indicator, at time zero; β_1_ estimates the change in mean value of each evaluation indicator per year during the pre-intervention period (as a baseline trend per year), namely slope pre-UMIS [28];β_2_ estimates the change in mean value of each evaluation indicator after the UMIS (the immediate effect of the UMIS on each evaluation indicator); and β_3_ estimates the changes in trend of the mean value of each evaluation indicator after the UMIS, compared with the trend before the UMIS, and included slope change or slope difference. The sum of β_1_ and β_3_ represents the post-UMIS slope. The error term e_*t*_ at time t represents the random variability not explained by the model. We used STATA software, version 12.0 (StataCorp LP, Texas USA), for estimation. The Breusch-Godfrey LM test [29] and Breusch-Pagan/Cook-Weisberg test [30] were used to test for the presence of autocorrelation and heteroskedasticity. The newey-west regression and robust regression in the Stata were used to correct the autocorrelation and heteroskedasticity.

Catastrophic payment rate (abbreviated as CPR) = (average annual out of pocket medical expenses / average annual disposable income of resident) * 100% [31]

A CPR exceeding 40% suggests that out of pocket (abbreviated as OOP) medical expenses in this specific year are not affordable for the resident [32]. Due to the limited availability of data, we used the average out of pocket medical expenses per hospitalization instead of the average annual out of pocket medical expenses of patients covered by UMIS. If the CPR exceeds 40%, based on the calculation of one hospitalization expense, we hypothesize that the CPR for a given patient within a given year must exceed 40%. We adjusted all expenditure data for inflation from 2002 through 2014, using the Chinese consumer price Index.

Average growth rate (abbreviated as AGR) = ((a_n_/a_1_)^1/n^-1))* 100%), a_n_ represents the value in the n year, a_0_ represents the value in the first year.

## Results

### Characteristics and trends of the universal medical insurance system in China

In China, the UMIS was initiated in 2008 and consisted of the Urban Employee’s Basic Medical Insurance (UEBMI), New Rural Cooperative Medical scheme (NRCMS), and Urban Resident Basic Medical Insurance (URBMI). Characteristics of the UMIS in 2014 are shown in Table 1. In 2014, the UMIS had covered 1.3 billion Chinese, and represented about 97.5% China’s total population, including urban and rural residents, compared to only 7.3% population for urban employees in 2002. In 2014, the average premium of UEBMI was up to 2841 RMB per capita compared to 383 RMB per capita in 1998. Moreover, the average premium of URBMI was 409 RMB per capita, including an average of 324 RMB government subsidies and 85 RMB of individual premium compared to 50 RMB (average 40 RMB government subsidies and 10 RMB of individual premium) in 2007. For NRCMS, the average premium per capita was roughly 411 RMB (an average of 320 governmental subsidies and 91 RMB of individual premium). Between 2002 and 2014, the average revenue and expenditure of UMIS funding was 383.456 billion RMB and 313.635 billion RMB, respectively.

**Table 1:** Characteristics of the UMIS in 2014.

Content	UEBMI	NRCMS	URBMI
**Year initiated**	1998	2003	2007
**Main target population** [33]	Urban employees (including private sector employees)	Rural residents (household unit)	Urban informal employees, students, children, elderly people without previous employment
**Population covered**	5.1 million (1998) [34] 283.3 million (2014)	80 million (2004) 736 million (2014)	42.9 million (2007) 314.5 million (2014)
**Risk-pooling**	At prefecture level	At county level	At prefecture level
**Covered cities**	333 municipalities	2854 counties	333 municipalities
**Enrollment feature**	Mandatory	Voluntary	Voluntary
**Benefit package**	Outpatient and inpatient medical service	Inpatient medical service	Inpatient and catastrophic outpatient medical service
**Source of funding** [35]	8% of annual payroll (6% from employers and 2% from employees)	Government subsidy and individual premium	Government subsidy and individual premium
**Annual premium per capita** [36]	383 RMB (1998) 2841 RMB (2014)	30 RMB (2003) 411 RMB (2014)	50 RMB (2007) 409 RMB (2014)
**Annual reimbursement ceiling** [33]	Six-times average wage of employee in the city	Six-times disposable income of local residents	Six-times income of local farmers
**Predominate payment method**	Global budget, capitation, diagnosis related groups (DRGs) [37]	Global budget, capitation, DRGs	DRGs, bed per day payment, capitation, global budget [38]
**Medical provider**	Enrolled hospitals	Enrolled hospitals	Enrolled hospitals

Note: UEBMI: Urban Employee’s Basic Medical Insurance; NRCMS: New Rural Cooperative Medical scheme; URBMI: Urban Resident Basic Medical Insurance.

**Fig 1:**
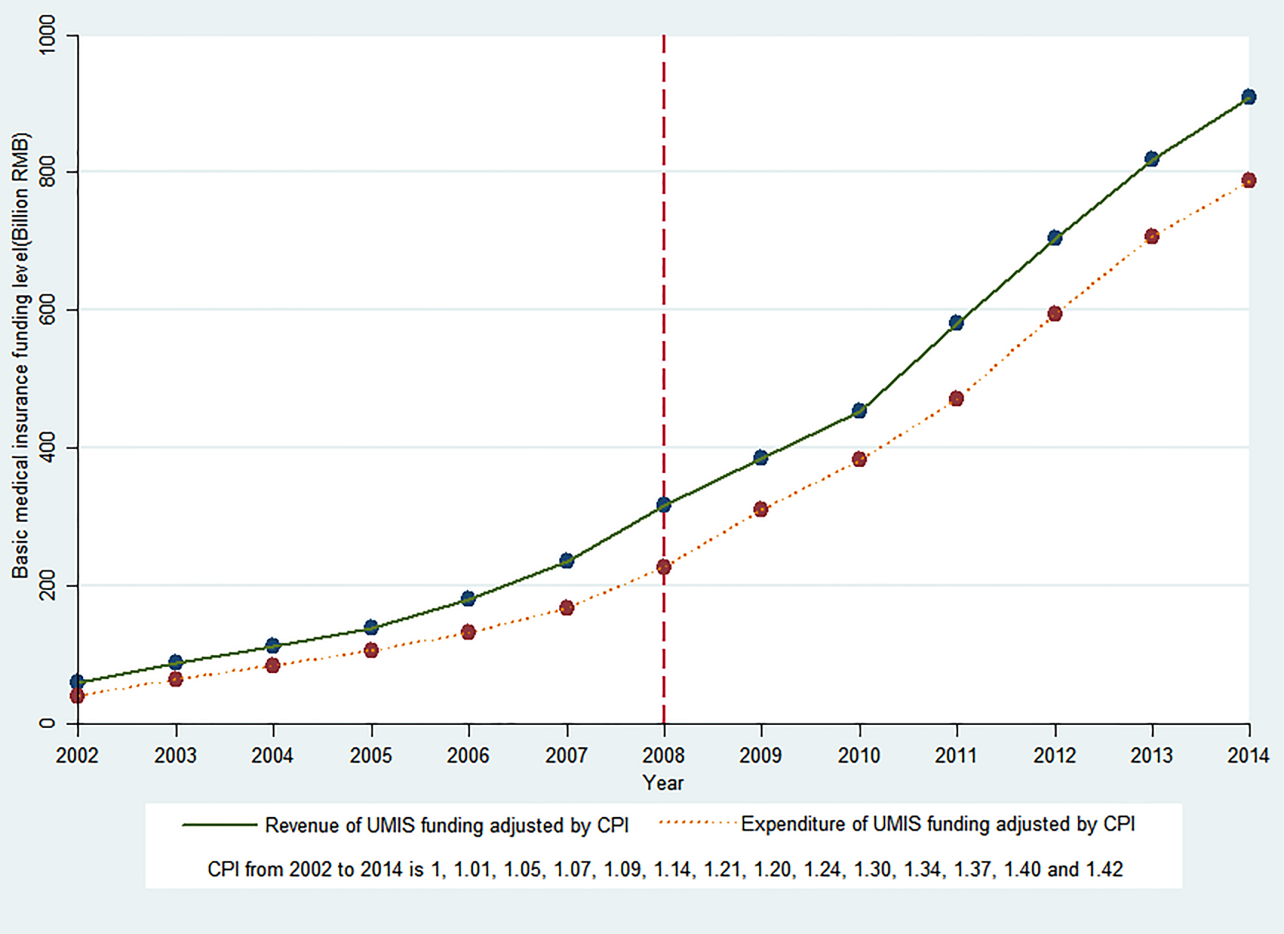
Trends in revenue and expenditure of UMIS funding in China.

Fig 1 presents the time series of revenue and expenditure of UMIS funding from 2002 to 2014. The revenue and expenditure of UMIS funding showed an increasing trend before and after the UMIS. Based on the STSR results, the trends of revenue and expenditure of UMIS funding were significantly increased (p<0.01) by 33.682 billion RMB revenue and 24.490 billion RMB expenditure per year before 2008. At initiation of the UMIS, the UMIS revenue showed an immediate decrease (coefficient = -37.034), and decreased by 37.034 billion RMB per year, however this was not statistically significant (p>0.1). Moreover, there was a significant and immediate decrease of expenditure of UMIS funding (coefficient = −46.912, p<0.05), which decreased by 46.912 billion RMB per year. After introduction of the UMIS, the annual trend increased in revenue (coefficient = 69.442) and expenditure (coefficient = 71.424), and was statistically significant (69.442 billion RMB more revenue per year and 71.424 billion RMB more expenditure per year compared to the pre-UMIS period (p <0.01). The STSR results are shown in Table 1 in S1 File. These data suggested that the trend increase in the expenditure of UMIS funding was higher compared to the increase in revenue of UMIS funding after 2008.

Current payment methods of medical insurance gradually changed from a simple payment by fee-for-service (FFS) to mixed payment methods, including capitation, global budgets, diagnosis-related groups (DRGs) and bed per day payment *etc* since several proposals were launched by Chinese government in 2011 and 2012. The three medical insurance schemes also defined enrolled hospitals before insurance payment. (Table 1)

### Changes of the quantity and quality of medical service supply before and after the UMIS

#### Licensed physicians, nurses, and hospital beds at the population level

Table 2 shows the estimated coefficients of the segmented regression model for licensed physicians and assistants, nurses, and hospital beds per 1000 individuals in urban and rural areas in China.

**Table 2:** Estimated coefficients of segmented regression for licensed physicians, nurses, and hospital beds per 1,000 individuals before and after the UMIS.

Coefficient	Number of licensed physicians and assistants per 1,000			Number of licensed nurses per 1,000			Number of hospital beds per 1,000	
	Overall	Urban	Rural	Overall	Urban	Rural	Overall	Rural^▲^
**β**_**0**_**(Intercept)**	1.056 ***	1.852 ***	0.926 ***	0.798 ***	1.092 ***	0.378 ***	2.164 ***	0.712 ***
**β**_**1**_ **(Slope pre- UMIS)**	0.018 ***	0.134 ***	0.060 ***	0.032 ***	0.225 ***	0.056 ***	0.076 ***	0.020 ***
**β**_**2**_ **(Level change after UMIS)**	0.007	-0.130 *	-0.080	-0.065 **	-0.229 **	-0.087 **	-0.056	0.085 ***
**β**_**3**_**(Slope change after UMIS)**	0.044 ***	0.006	-0.018	0.112 ***	0.068 ***	0.039 **	0.219 ***	0.043 ***
**β**_**1**_**+β**_**3**_ **(Slope post- UMIS)**	0.062	0.140	0.042	0.144	0.293	0.095	0.294	0.063

Significant codes (P value of two-sided test): <0.01‘***’, <0.05‘**’, <0.1‘*’.

‘^▲^’: The number of beds in the township hospitals per 1000 agricultural population.

Regarding the number of licensed physicians and assistants (abbreviated as physicians), a significant increase was observed (coefficient = 0.018, p<0.01), 0.018 physicians per 1000 people every year before the UMIS. When the UMIS was initiated in 2008, the level change in average number of physicians per 1000 individuals showed a slightly increase (coefficient = 0.007), which increased by 0.007 physicians per 1000 individuals per year but was not statistically significant (p>0.1). After introduction of the UMIS, the increased trend was statistically significant (p<0.01), 0.044 physicians per 1000 individuals per year more when compared to the pre-UMIS period. In both urban and rural areas, the average number of physicians per 1000 individuals significantly increased every year before the UMIS (p<0.01). After the UMIS, an increasing trend in the average number of urban physicians per 1000 individuals was not statistically significant but showed a significant decrease to the level of urban physicians (p<0.1). Both a slow increasing trend and a decreased immediate level in the average number of rural physicians per 1000 individuals were not statistically significant.

Regarding the number of nurses per 1000 individuals, at the overall and urban and rural level, similar trends and significant changes were observed before and after the UMIS. Prior to the UMIS, there was a significant increase in the average number of nurses per 1000 individuals (p<0.01). After the UMIS, however, an immediate and significant decrease in level change (p<0.05) and a significant ascending trend in the average number of nurses were observed compared to the pre-UMIS period (p<0.01).

For the number of hospital beds per 1000 individuals, a significant increase in the average number of overall hospital beds per year was found before the UMIS (p<0.01). However, after the UMIS, an immediate decrease in this level was observed but this was not statistically significant (p>0.1). In addition, a significantly increasing trend was found when compared to the pre-UMIS period (p<0.01). The trend and level change in the average number of beds in township hospitals per 1000 individuals of the agricultural population showed a significant rise (p<0.01) before and after the UMIS. As shown in Table 2, the numbers of physicians, nurses, and hospital beds per 1000 individuals showed an ascending trend before and after the UMIS.

#### Number of outpatient visits and inpatient visits at the hospital level

**Fig 2:**
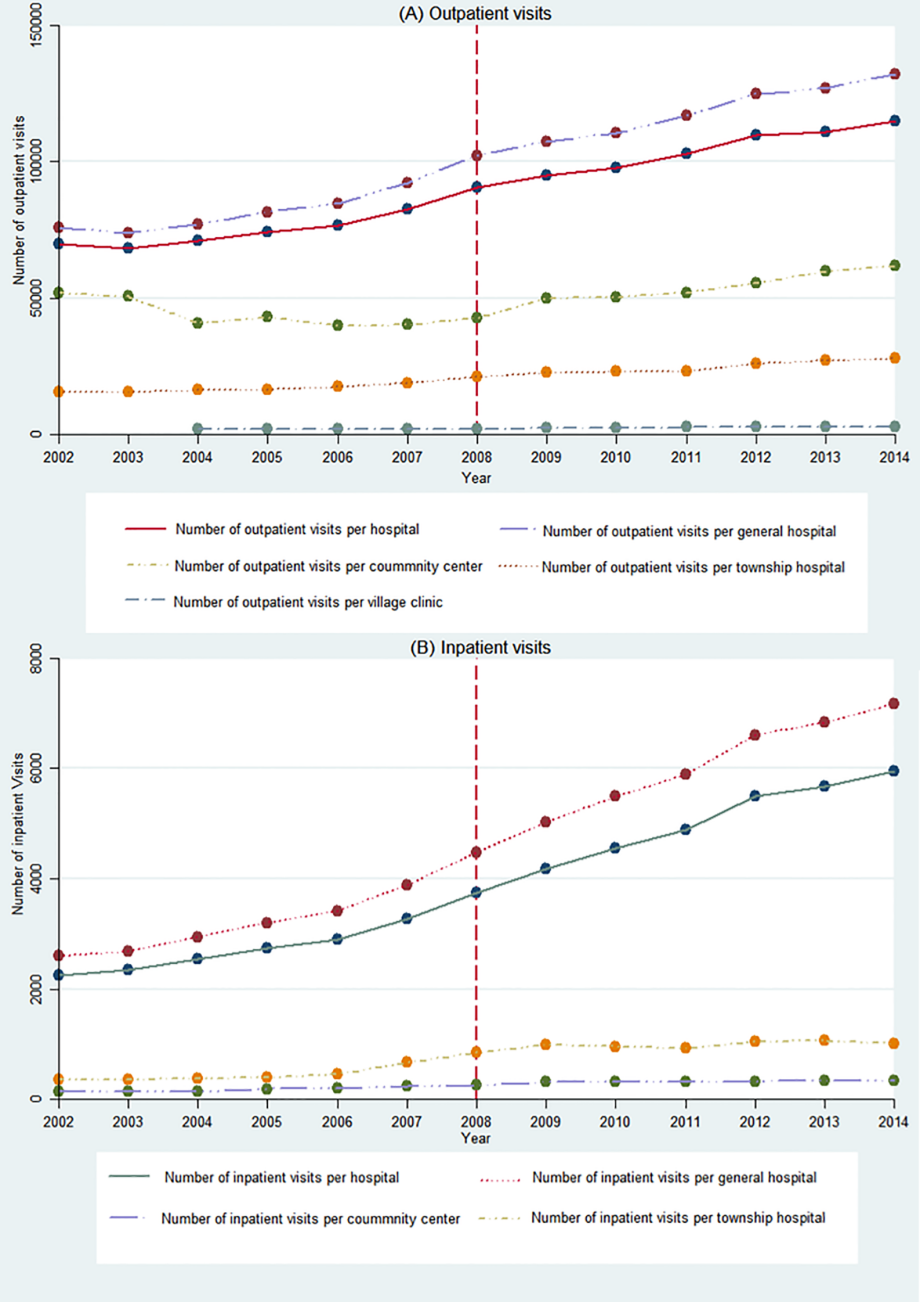
Trends in outpatient and inpatient visits in the different-level hospitals. (A) Outpatient visits. (B) Inpatient visits.

Fig 2 shows the time series of outpatient and inpatient visits in the different-level hospitals between 2002 and 2014. Overall, there was an increasing trend in hospital visits in the different hospitals. Table 3 displays the estimated STSR coefficients for outpatient and inpatient visits in the different-level hospitals.

**Table 3:** Estimated coefficients of segmented regression for hospital outpatient and inpatient visits before and after the UMIS.

Coefficient	Overall hospital		General hospital		Community center		Township hospital		Village clinics
	Out patient	In patient	Out patient	In patient	Out patient	In patient	Out patient	In patient	Out patient
**β**_**0**_**(Intercept)**	64467 ***	1971 ***	68992 ***	2230 ***	53190 ***	108 ***	14593 ***	255 ***	2133 ***
**β**_**1**_ **(Slope pre- UMIS)**	2625 ***	199 ***	3391 ***	254 ***	-2512 ***	20 ***	635 ***	52 ***	16
**β**_**2**_ **(Level change after UMIS)**	5880 **	260 **	7496 **	341 **	3333	43 *	1518 *	304 ***	-94
**β**_**3**_ **(Slope change after UMIS)**	1601 **	176 ***	1705 **	203 ***	5451 ***	-8	507 **	-26	130 ***
**β**_**1**_**+β**_**3**_**(Slope post- UMIS)**	4225	375	5096	457	2938	12	1142	26	146

Significant codes (P value of two-sided test): <0.01‘***’, <0.05‘**’, <0.1‘*’.

Prior to the UMIS, the number of outpatient visits in overall hospitals, general hospitals, and township hospitals all showed a significant increasing trend (p<0.01). In addition, the number of outpatient visits in village clinics also showed an ascending trend, however this trend was not statistically significant (p>0.1). Moreover, the number of outpatient visits in the community centers showed a significant decreasing trend (coefficient = -2512, p<0.01). After start of the UMIS, there was a significant and immediate increase in level change for the average number of outpatient visits in overall hospitals, general hospitals, and township hospitals. However, no significant differences were found before and after the UMIS regarding the level of change of outpatient visits in community centers and village clinics. The annual increasing trends of outpatient visits in the five-level hospitals were all significant (p<0.01). In addition, the increasing trend of outpatient visits per year in community centers was the greatest, including 5451 outpatient visits more per year when compared to the pre-UMIS period (coefficient = 5451, p<0.01).

The numbers of inpatient visits per year in the four-level hospitals all showed a significantly increasing trend (p <0.01) when compared to the pre-UMIS period. After start of the UMIS, there was a significant immediate increase in level change for inpatient visits in the four-level hospitals (p<0.1). The increasing trend of inpatient visits per year in overall hospitals and general hospitals was statistically significant (p<0.01). However, the increasing trend of annual inpatient visits in community centers and township hospitals was reduced but not statistically significant. The trend increase of annual inpatient visits in general hospitals was the greatest, and included 203 inpatient visits more per year when compared to the pre-UMIS period (coefficient = 203, p<0.01).

#### Fatality rate of inpatients at the hospital level

**Fig 3:**
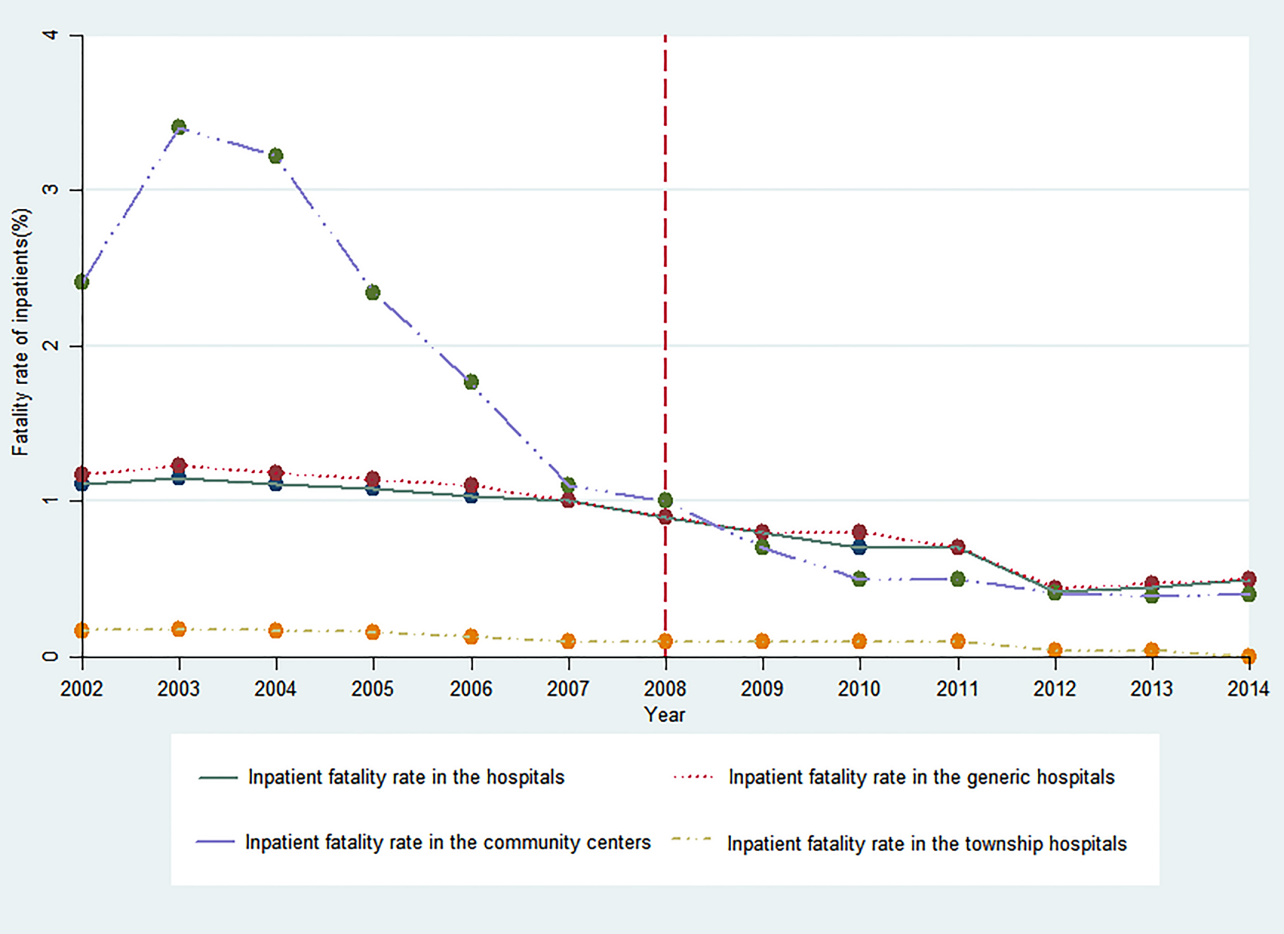
Trends in the fatality rates of inpatients in the different-level hospitals.

Fig 3 and Table 4 show the trends in the fatality rate of inpatients in different-level hospitals and the STSR results. Before the UMIS, fatality rates of inpatients in four-level hospitals all showed a significantly decreasing trend (p<0.1). After initiation of the UMIS, no significant level changes in average fatality rates of inpatients in the four-level hospitals (P>0.05) were observed. The decreases in trend of the average fatality rates of inpatients in the overall hospital and general hospital were significant (p<0.01, p<0.1), whereas the decreasing trend was not significant in community center and township hospitals compared to the pre-UMIS period.

**Table 4:** Estimated coefficients of segmented regression for the fatality rate of inpatients before and after the UMIS.

Coefficient	Overall hospital	Generic hospital	Community center	Township hospital
**β**_**0**_ **(Intercept)**	1.174 ***	1.265 ***	3.607 ***	0.203 ***
**β**_**1**_**(Slope pre- UMIS)**	-0.027 ***	-0.037 **	-0.353 *	-0.015 ***
**β**_**2**_**(Level change after UMIS)**	-0.063	-0.070	-0.574	0.022
**β**_**3**_ **(Slope change after UMIS)**	-0.051 ***	-0.043 *	0.263	-0.003
**β**_**1**_**+β**_**3**_**(Slope post- UMIS)**	-0.078	-0.079	-0.090	-0.017

Significant codes (P value of two-sided test): <0.01‘***’, <0.05‘**’, <0.1‘*’.

The STSR results for the fatality rates of the eight diseases mentioned above in generic hospitals are shown in Table 2 in S1 File. Prior to the UMIS, the fatality rates of eight diseases all showed a declining trend. Except for the AMI, the fatality rates of the other seven diseases showed a significant decrease. After the UMIS, there was a significant and immediate increase in the change in fatality rate of heart failure (coefficient = 1.597, p<0.01). The fatality rate of AMI showed a significant decreasing trend (coefficient = −0.835, p<0.01), which showed a 0.835% lower AMI fatality rate per year compared to the pre-UMIS period. For the other seven diseases, no significant changes in the trend of fatality rate per year were found. As shown in Table 4 and Table 2 in S1 File, the fatality rates of inpatients and the eight diseases per year all showed a declining trend before and after the UMIS.

### Changes of catastrophic payment rates of UMIS beneficiaries

The average medical expenses per hospitalization of UMIS inpatients in the enrolled hospitals showed a rising trend from 2008 to 2014. The average medical expenses of UEBMI inpatients were 5703.28 RMB (CPI adjustment) in 2008, and 7742.78 RMB in 2014, about 5.23% average growth rate (AGR). The OOP expenses of UEBMI inpatients ranged from 1927.14 RMB in 2008 to 2129.26 RMB (1.68% AGR) in 2014. The average medical expenses and OOP expenses of URBMI inpatients were 3740.1RMB and 2318.46RMB in 2008, and 5363.79 RMB (6.19% AGR) and 2427.65 RMB (0.77% AGR) in 2014 respectively. The average medical expenses and OOP expenses of NRCMS inpatients were 2321.86 RMB and 1439.56 RMB in 2008, and 3359.32 RMB (6.35% AGR) and 1545.29 RMB (1.19% AGR) in 2014 respectively. The average medical expenses of total UEBMI inpatients from 2008 to 2014 were the highest (6959.69 RMB per hospitalization), whereas for NRCMS (2885.77 RMB per hospitalization) expenses were the lowest. The URBMI inpatients showed the highest average OOP expenses per hospitalization (2326.33 RMB) versus 2058.49 RMB for UEBMI inpatients and 1477.15 RMB for NRCMS inpatients. Furthermore, the inpatients enrolled in the UEBMI system had a much higher average reimbursement rate (70.26%) than those enrolled in URBMI (50.98%) or NRCMS (48.00%).

With the rapid growth of income and UMIS investment, the affordability of medical expenses for patients covered by UMIS is changing simultaneously. Although the OOP medical expenses increase, patients’ medical expenses become more affordable. As shown in Table 5, the average catastrophic payment rate (CPR) of inpatients covered by UMIS showed an obvious declining (AGR from -5.29% to -9.45%) from 2008 to 2014. From low to high income levels, the CPRs of UEBMI inpatients were all the lowest (31.01% to 5.78%), corresponding to the CPRs of NRCMS inpatients from 95.08% to 11.88%. For low-income patients, the average CPRs under three insurance schemes were all over 30%. For high-income patients, the average CPRs under three insurance schemes were about 10%. For middle-income patients, only the CPR under the NRCMS remained higher than 30%. However, the average decrease of CPR for NRCMS inpatients was the lowest. This suggests that the CPR for NRCMS patients is the highest.

**Table 5:** Rates of catastrophic payment of UMIS inpatients in three income levels from 2008 to 2014.

Year	20% Lowest population (%)			20% Middle population (%)			20% Highest population (%)		
	UEBMI	URBMI	NRCMS	UEBMI	URBMI	NRCMS	UEBMI	URBMI	NRCMS
**2008**	38.25	46.02	115.73	16.62	19.99	41.30	6.70	8.06	15.37
**2009**	35.12	49.39	113.51	15.34	21.57	39.06	6.31	8.87	14.28
**2010**	34.39	36.09	102.76	15.19	15.93	36.80	6.36	6.67	13.68
**2011**	28.80	32.12	101.69	12.95	14.45	32.77	5.38	6.00	12.12
**2012**	28.55	27.37	80.53	13.18	12.64	26.49	5.74	5.51	9.81
**2013**	25.38	27.13	73.20	11.84	12.65	23.81	5.15	5.50	8.89
**2014**	26.57	30.29	78.14	11.18	12.75	22.76	4.84	5.52	9.03
**AVG**	31.01	35.49	95.08	13.76	15.71	31.86	5.78	6.59	11.88
**AGR**	-5.90	-6.73	-6.34	-6.39	-7.22	-9.45	-5.29	-6.14	-8.48

Note: N/A: data not available. AVG: average. AGR: average growth rate.

In tertiary hospitals, the OOP medical expenses of UEBMI inpatients ranged from 3101.76 RMB in 2010 to 2840.41 RMB (-2.18% AGR) in 2014 compared to 3655.64 RMB in 2010 to 3704.19 RMB (0.33% AGR) in 2014 for URBMI inpatients. In first-level hospitals, the OOP expenses of UEBMI inpatients ranged from 814.87 RMB in 2010 to 879.02 RMB (1.91% AGR) in 2014 compared to 776.20 RMB in 2010 to 661.42 RMB (-3.92% AGR) in 2014 for URBMI inpatients. For NRCMS inpatients, the OOP expenses in county hospitals ranged from 4700.22 RMB in 2010 to 4335.16 RMB (-2.00% AGR) in 2014, compared to 347.17 RMB in 2010 to 260.43 RMB (-6.94% AGR) in 2014 in township hospitals. The CPRs of three medical insurance schemes for patients with three income levels all showed a downward trend at three levels of hospitals. However, it is clear that for low-income inpatients, even with reimbursement of UMIS, the average OOP medical expenses of a single hospitalization in a tertiary hospital is higher than the disastrous payments (average CPR> 40%), especially for the rural poor (average CPR>250%). For a middle-income patient with UEBMI, catastrophic payments can be reached with two hospitalizations per year in tertiary hospitals, compared to over four hospitalizations per year for high-income patients.

### Changes on seeking-care choice of UMIS beneficiaries

Table 6 shows the average rates of seeking-care choice of UMIS inpatients for different-level hospitals ranging from 2008 to 2014. Among three levels of hospitals, most UEBMI inpatients (48.34%) sought medical care in tertiary hospitals. Correspondingly, 43.39% of URBMI inpatients were more inclined to choose secondary hospitals. And 41.85% of NRCMS inpatients would like to seek medical treatment in township hospitals. From 2008 to 2014, the tertiary hospitals, first-level hospitals and county hospitals had the highest growth rate of seeking-care choice for UEBMI and URBMI inpatients, as well as NRCMS inpatients respectively. Compared to URBMI and NRCMS beneficiaries, UEBMI beneficiaries were more willing to choose the tertiary hospitals for medical services. UMIS beneficiaries showed the intention of growth to seek hospitalization services in tertiary hospitals or outside county hospitals with a higher level.

**Table 6:** Rates of seeking-care choice of UMIS inpatients for three levels of hospitals from 2008 to 2014.

Year	UEBMI			URBMI			NRCMS		
	Ter-Hos (%)	Sec-Hos (%)	Fir-Hos (%)	Ter-Hos (%)	Sec-Hos (%)	Fir-Hos (%)	Out-Hos (%)	Cou-Hos (%)	Tow-Hos (%)
**2008**	N/A	N/A	N/A	N/A	N/A	N/A	16.62	33.25	50.13
**2009**	43.94	40.2	15.01	41.46	47.64	10.09	17.1	34.24	48.66
**2010**	43.28	42.98	13.74	30.72	53.34	15.94	18.74	36.85	44.41
**2011**	45.58	35.79	18.63	36.07	45.52	18.41	20.99	39.69	39.32
**2012**	49.25	34.47	16.29	39.62	36.6	24.77	20.57	40.5	38.93
**2013**	54.3	33.72	11.98	41.47	38.7	19.83	20.94	42.04	37.02
**2014**	53.68	34.67	11.56	44.35	38.52	17.13	21.87	43.68	34.45
**AVG**	48.34	36.97	14.54	38.95	43.39	17.70	19.55	38.61	41.85
**AGR**	4.10	-2.90	-4.90	1.40	-4.20	9.50	4.10	4.20	-5.60

Note: Ter-Hos: Tertiary hospital; Sec-Hos: Secondary hospital; Fir-Hos: First level hospital; Out-Hos: Outside county hospital; Cou-Hos: County hospital; Tow-Hos: Township hospital; N/A: data not available.

## Discussion

Our study describes the overview changes on the accessibility of medical service supply and affordability and seeking hospital-care choice of patients after the start of the UMIS in 2008. The main findings of the study are presented in the following five aspects:

Firstly, the increasing trends of revenue and expenditure of UMIS funding per year significantly accelerated after introduction of the UMIS in 2008. Moreover, the increase in trend of expenditure was higher compared to that of the increase in revenue after 2008 compared to that prior to 2008. In 2014, the UMIS covered over 97.5% of the entire population in China. From the beginning of the transformation of basic medical insurance in 1998 to 2014, China has achieved universal medical insurance coverage for almost the entire population. Our results therefore support many previous findings, indicating the successful expansion of insurance coverage [5, 10, 39, 40].

Secondly, the accessibility of medical services was significantly improved after start of the UMIS. At the population level, there were statistically significant increases in the trend of overall physicians, nurses, and hospital beds per 1000 individuals after start of the UMIS when compared to the pre-UMIS period. The increasing trend in the average number of physicians in rural areas per 1000 individuals showed a non-significant decrease. At the hospital level, the increasing trends of outpatient visits per year in the five-level hospitals were all significantly increased after start of the UMIS. For inpatient visits per year, only overall hospitals and general hospitals showed a statistically significant increasing trend compared to the pre-UMIS period. However, inpatient visits in community centers and township hospitals was not significantly increased compared to the pre-UMIS period. This indicated that the utilization level of medical services was improved, and that a preference was given to hospitalization service.

In addition, with regard to the utilization level of medical services, other studies also have given some inconsistent findings. Zhou et al. using cross-sectional data from the 4th national health services survey of the Shaanxi province found that UEBMI enrolled patients significantly increased outpatient visits and hospitalization compared to the uninsured. However, the effect of URBMI was limited given that none of the improvement of health services utilization with statistical significance compared with the uninsured [15]. Chen et al. using four-wave URBMI survey (2008–2011) panel data found that URBMI significantly increased both the inpatient and outpatient services utilization [14]. Regarding NRCMS, studies from Wagstaff et al., You et al., Zhang el al., and Zou et al. confirmed that NRCMS had substantially improved outpatient and inpatient utilization among participants. Babiarz et al. surveyed 8339 participants in 160 village primary care clinics across five Chinese provinces in 2004 and 2007. They found that NRCMS was associated with a 26% increase in weekly patient flow in village clinics. NRCMS participants were associated with a 5% increase in village clinic use [41–44].

Thirdly, we found that the quality of overall medical services was improved after initiation of the UMIS. The fatality rates of inpatients at four-level hospitals and the eight diseases mentioned above in general hospitals per year showed a declining trend both before and after the UMIS. After the UMIS, the declining trend of the average fatality rate of inpatients in the overall hospitals and general hospitals was significantly increased, whereas the decreases in community centers and township hospitals was not significantly different when compared to the pre-UMIS period. Only the fatality rate of AMI was significantly decreased in trend. Overall, the life expectancy of the Chinese increased from 71.00 in 2002, 74.00 in 2008, to 75.55 in 2014 [45].

As early in 1988, a study by Davis C et al. reported the impact of DRGs on the quality of health care in the US. Under the prospective payment system, they found the DRGs could improve the quality of inpatient care by discouraging unnecessary and potentially harmful procedures, and by encouraging the concentration of complex procedures in facilities. And the mortality rate and the rate of readmission of inpatients didn’t increase [46]. Winnie Yip, et al empirically evaluated the impact of a change of provider payment (from FFS payment to prospective payment) in January 1997 in Hainan province, China. They found that the prepayment slowed down the increase in spending on expensive drugs and high technology services, compared to FFS [47]. At present, there are very few reports about the impact of the insurance payment on the quality of medical services in China. With the advancement of the DRGs and the global budget in China, further research is needed on the effects of medical insurance payments for medical quality.

Fourthly, we provided the evidence that the affordability of medical expenses per hospitalization for inpatients enrolled in UMIS was effectively improved, although the OOP medical expenses per hospitalization increased from 2008 to 2014. The CPRs for UMIS inpatients with three income levels were declining at both the overall level and hospital level. The patients with different medical insurance schemes have different insurance reimbursement rates according to the local policy. Generally, UEBMI can provide a more generous reimbursement for the beneficiaries than that of URBMI and NRCMS. Whichever income level, the CPR for UEBMI beneficiaries was the lowest. At the same time, primary medical institutions also have a higher reimbursement than the higher level hospitals. So the expenses resulting from a single hospitalization for a low-income inpatient in a tertiary hospital likely result in catastrophic payments. NRCMS patients are more likely to fall into catastrophic payments than recipients of UEBMI and URBMI, especially for the rural poor. Therefore, the differences in patients’ affordability are mainly due to differences in medical insurance schemes and differences in selection of hospitals. This inequality of current UMIS exacerbates the affordability gap between people in different income levels. Our evidence also supported previous findings that its ability to withstand catastrophic medical expenses (CMEs) for the poor was still relatively weak.

A study by Li et al. showed that among 55,556 households from the Fourth National Health Service Survey of China in 2008, the rate of CME was 13.0% and that of impoverishment was 7.5%. Rates of CME were higher among households in which members were hospitalized or in households from rural or poorer regions. Families enrolled in the UEBMI and URBMI had lower rates of CME than those enrolled in the NRCMS [48]. A study by Meng et al. showed that CMEs in poor households occurred twice as much compared to wealthier households in China, and this gap did not change from 2003 to 2011 [10]. Cui et al. showed that OOP rates of urban employee and residents were about 29% and 49%, respectively, according to data from the National Sample Survey on Medical Service Utilization of Basic Medical Insurance participants in 2009–2012. However, the individual burden for inpatients with expenses above US $3,200 had not been alleviated enough [49]. Babiarz et al. demonstrated that, from 2004 to 2007, OOP of NRCMS participants fell by 19% and financial risk declined by 24–63% in village clinics. NRCMS provides some financial risk protection for individuals in rural China. Ma et al. surveyed 1,525 households covered by NRCMS in the Zhejiang, Hubei, and Chongqing provinces in 2011 and found even after NRCMS reimbursement in all three provinces, the medical financial burden and the prevalence of CME were both remarkably high for low income rural residents in China [50]. Liu et al. demonstrated that poor people benefit less from NRCMS in terms of health service utilization in China [51]. Yang et al. selected 41,037 individuals covered by NRCMS based on the data of 5th National Health Service Survey of Shaanxi Province in 2013 and found NRCMS could alleviate health payment-induced poverty [52].

Lastly, medical insurance plays an important role in guiding patient’s flow of seeking-care in the different level hospitals. We found that a higher proportion of UEBMI patients were inclined to choose inpatient services in tertiary hospitals and URBMI patients tended to choose secondary hospitals, however, NRCMS patients preferred to township hospitals. Moreover, the trend of seeking hospital-care choices of patients enrolled in the UEBMI, URBMI, and NRCMS for tertiary hospitals increased from 2008 to 2014. These seeking hospital-care choices of UEBMI patients for secondary and first-level hospitals and URBMI patients for secondary hospitals, as well as NRCMS patients for township hospitals, show downward trends. Therefore, it is believed that most beneficiaries of UMIS are more willing to be hospitalized at higher levels of hospitals.

The question remains, what should we do to improve the accessibility of high-quality medical services and the level on economic risk resilience for beneficiaries of the UMIS?

Our study demonstrates that the accessibility of medical services has been improved after the UMIS. At the hospital level, the inpatient visits in the general hospitals was greatest increase in the trend, while the increasing trends in the community center and township hospitals were reduced. In addition, UMIS beneficiaries showed a growing preference to choose the hospitalization services in the tertiary hospitals. Therefore, currently patients to access the medical services in the top-level hospitals are still difficult. It is also difficult for patients to obtain a substantial reduction of medical costs in China.

A variety of studies put forward a meaningful explanation and solution for these questions. The study by Liu et al. found that social medical insurance participation had a weak negative or no significant association with the OOP of hospitalized patients. This result was obtained from the lack of management behavior and purchasing mechanisms of social medical insurance, resulting in undermining the function of the reimbursement mechanism and mitigating the association between insurance participation and OOP [53]. Barber and Yao hypothesized that promoting utilization at the primary level requires shifting qualified human resources and technology to the primary level and increasing quality of care, particularly for the management of chronic conditions that require more qualified staff and stronger referral systems [54]. In order to reduce the incidence rate of CME for the poor, Pan et al. stressed that rural health institutions should strengthen capacity-building. The integration of urban and rural insurance schemes should be coordinated with ongoing efforts to establish referral mechanisms among different levels of care. Integrated insurance schemes can also benefit the poor in both urban and rural areas in China [11]. Li et al. believed that policy-makers should focus on designing improved insurance plans by expanding the benefit package, redesigning cost sharing arrangements and provider payment methods and developing more effective expenditure control strategies [48]. Meng et al. put forward that achievement of universal health coverage in China needed systemic strategies including consolidation of the social medical insurance schemes to benefit rural migrants, elderly people and those with non-communicable diseases. Combined with the universal medical insurance experience of Thailand, Li et al. suggested that China should improve aspects, such as reducing the gaps in the benefit package across different schemes, changing the fee-for-service payment system, strengthening the primary health care referral system in coordination with the transformation of the medical insurance system and integrating fragmented insurance schemes to raise the risk pooling level [55].

## Conclusions

The UMIS was introduced in 2008. In 2014, the UMIS had covered 97.51% of the entire population in China. Indeed, China achieved universal medical insurance coverage for almost the entire population. After introduction of the UMIS in 2008, the annual increasing trend of average revenue and expenditure of UMIS funding was significantly increased. Implementation of the UMIS contributed to the increase of physicians, nurses, and hospital beds and the level of hospital outpatient and inpatient visits. Moreover, the quality of overall medical services was also improved. In general hospitals, the fatality rates of inpatients and the eight diseases mentioned above per year showed a declining trend before and after the UMIS. In addition, the declining trend of the fatality rate of inpatients in the hospital was increased. In 2014, the life expectancy of the Chinese improved. However, there were no significant and prospective changes in trends of physicians in rural areas per 1000 individuals, inpatient visits and inpatient fatality rate in community centers and township hospitals compared to the pre-UMIS period.

At the same time, the affordability of the beneficiaries of UMIS for medical expenses was successfully ameliorated after the UMIS. The rates of catastrophic payments for UMIS inpatients at different income levels are declining at three levels of hospitals. Whichever income level, the rate of catastrophic payments for inpatients of Urban Employee’s Basic Medical Insurance is the lowest. For low-income people, hospitalization costs of a tertiary hospital can lead to catastrophic payments. UMIS beneficiaries also showed the intention of growth to seek hospitalization services in tertiary hospitals.

Therefore, in order to further guarantee people access to lower costs and high-quality medical services, the Chinese government should reform the value-based payment of medical insurance to promote the integration of medical services and the formation of a tiered treatment system. Finally, medical insurance institutions may promote the integration of three medical insurance schemes and thereby expand the risk pool of funds, as well as the establishment of supplementary medical insurance packages of serious illness for the poor to enhance affordability of disease treatment.

## Limitation of the study

In this study, we mainly used the average-level data of medical service institutions and average hospital service utilization data of the beneficiaries of three basic medical insurance schemes, rather than individual level data. Therefore, the average medical expenses and OOP expenses per hospitalization did not represent the actual costs of a single patient covered by UMIS but for the overall level. In addition, the study results did not consider the regional differences of medical expenses and reimbursement levels of medical insurance.

Due to the limited data of three medical insurance schemes before and after the UMIS, we did not discuss the mutual relationships between the utilization improvement of medical services and death decrease and affordability increase of UMIS beneficiaries. At the same time, the synergistic effects of other health policies, such as an essential medicine policy launched in 2009, and internal management measures of different hospitals on patient’s accessibility, quality and affordability of medical services were not considered.

## Supporting information

S1 File The results of segmented regression model for UMIS funding and fatality rates of eight diseases before and after the UMIS. (DOC)

S2 File Original data in the study. (DOCX)

## References

[1] Filipski MJ, Zhang Y, Chen KZ. Making health insurance pro-poor: evidence from a household panel in rural China. BMC HEALTH SERV RES, 2015;15(1):1–13. doi: 10.1186/s12913-015-0871-7 26017455 10.1186/s12913-015-0871-7 PMC4446963

[2] Liu Y. Reforming China’s urban health insurance system. HEALTH POLICY, 2002;60(2):133–50. doi: 10.1016/S0168-8510(01)00207-X 11897373 10.1016/s0168-8510(01)00207-x

[3] Fang P, Dong S, Xiao J. The Institutional Change and Approach Choice of Governmental Health Input in China. Wuhan University Journal (Philosophy & Social Sciences), 2009;62(2):201–12. (Chinese)

[4] Tian X. Reflections on the Establishment of China’s Medical Insurance System. Chinese Health Economics, 1990;09(05):11–3. (Chinese)

[5] Yu H. Universal health insurance coverage for 1.3 billion people: What accounts for China’s success? HEALTH POLICY, 2015;119(9):1145–52. doi: 10.1016/j.healthpol.2015.07.008 26251322 10.1016/j.healthpol.2015.07.008 PMC7114832

[6] Guo J. The Development and Discussion of Public Medical Care Reform. Reform & Openning, 2016(18):30, 32. (Chinese)

[7] National Health And Family Planning Commission, China. Analysis Report on the Third National Health Services survey of China in 2003. [Internet]. 2003 http://www.nhfpc.gov.cn/cmsresources/mohwsbwstjxxzx/cmsrsdocument/doc9909.pdf.

[8] Wang B. Medical insurance payments are different from financial subsidies. China Social Security, 2016(05):82. (Chinese)

[9] Xu L, Wang Y, Collins CD, Tang S. Urban health insurance reform and coverage in China using data from National Health Services Surveys in 1998 and 2003. BMC HEALTH SERV RES, 2007;7(1):1–14. doi: 10.1186/1472-6963-7-37 17335584 10.1186/1472-6963-7-37 PMC1828155

[10] Meng Q, Xu L, Zhang Y, et al Trends in access to health services and financial protection in China between 2003 and 2011: a cross-sectional study. LANCET, 2012;379(9818):805–14. doi: 10.1016/S0140-6736(12)60278-5 22386034 10.1016/S0140-6736(12)60278-5

[11] Pan XF, Xu J, Meng Q. Integrating social health insurance systems in China. LANCET, 2016;387(10025):1274–5. doi: 10.1016/S0140-6736(16)30021-6 10.1016/S0140-6736(16)30021-6 27025430

[12] Liang X, Guo H, Jin C, Peng X, Zhang X. The effect of new cooperative medical scheme on health outcomes and alleviating catastrophic health expenditure in China: a systematic review. PLOS ONE, 2012;7(8):e40850 doi: 10.1371/journal.pone.0040850 22916098 10.1371/journal.pone.0040850 PMC3423411

[13] Zhang L, Li S, Yi H, D’Intignano LM, Ding Y. Correlation Between New Cooperative Medical Scheme Policy Design and Catastrophic Medical Payment: Evidence From 25 Counties in Rural China. ASIA-PAC J PUBLIC HE, 2015;28 doi: 10.1177/1010539515612907 26512031 10.1177/1010539515612907

[14] Chen G, Liu GG, Xu F. The impact of the urban resident basic medical insurance on health services utilisation in China. PHARMACOECONOMICS, 2014;32(3):277–92. doi: 10.1007/s40273-013-0097-7 24178373 10.1007/s40273-013-0097-7

[15] Zhou Z, Zhou Z, Gao J, et al The Effect of Urban Basic Medical Insurance on Health Service Utilisation in Shaanxi Province, China: A Comparison of Two Schemes. PLOS ONE, 2014;9(4):e94909 doi: 10.1371/journal.pone.0094909 eCollection 2014. 24740282 10.1371/journal.pone.0094909 PMC3989255

[16] Lancet editor. What can be learned from China’s health system? LANCET, 2012;379(9831):777 doi: 10.1016/S0140-6736(12)60874-5 22656868 10.1016/S0140-6736(12)60874-5

[17] European Commission EU. Health interventions: health services (European Core Health Indicators). [Internet].2017 https://ec.europa.eu/health/indicators/indicators_en.

[18] Copnell B, Hagger V, Wilson SG, Evans SM, Sprivulis PC, Cameron PA. Measuring the quality of hospital care: an inventory of indicators. INTERN MED J, 2009;39(6):352–60. doi: 10.1111/j.1445-5994.2009.01961.x 19323697 10.1111/j.1445-5994.2009.01961.x

[19] Agency For Healthcare Research And Quality US. Fact Sheet on Inpatient Quality Indicators. [Internet].2017 https://www.ahrq.gov/sites/default/files/wysiwyg/professionals/systems/hospital/qitoolkit/combined/a1a_combo_iqifactsheet.pdf.

[20] Centers For Medicare Medicaid Services US. Hospital Quality Alliance (HQA) 2004–2007 Measure Build Out Table. [Internet]. 2006 https://www.cms.gov/Medicare/Quality-Initiatives-Patient-Assessment-Instruments/HospitalQualityInits/downloads/HospitalHQA2004_2007200512.pdf.

[21] Organisation For Economic Co-operation And Development. Healthcare quality indicators. [Internet]. 2017 https://ec.europa.eu/health/indicators/other_indicators/quality_en.

[22] GBD 2015 Healthcare Access and Quality Collaborators.Healthcare Access and Quality Index based on mortality from causes amenable to personal health care in 195 countries and territories, 1990–2015: a novel analysis from the Global Burden of Disease Study 2015. LANCET, 2017;390(10091):231–66. doi: 10.1016/S0140-6736(17)30818-8 28528753 10.1016/S0140-6736(17)30818-8 PMC5528124

[23] National Bureau of Statistics, China. China Statistical Yearbooks from 2003 to 2015. [Internet]. 2016 http://www.stats.gov.cn/tjsj/ndsj/.

[24] National Health And Family Planning Commission, China. Yearbooks of China Health and Family Planning, 2014 and 2015. First Edition ed Beijing, China: Pecking Union Medical College Press, 2015.

[25] National Health And Family Planning Commission, China. China Health Statistics Yearbooks from 2003 to 2013. [Internet]. 2016 http://www.nhfpc.gov.cn/zwgkzt/tjnj/list.shtml.

[26] Zhang Y, Donohue JM, Lave JR, O’Donnell G, Newhouse JP. The effect of Medicare Part D on drug and medical spending. N Engl J Med, 2009;361(1):52–61. doi: 10.1056/NEJMsa0807998 19571283 10.1056/NEJMsa0807998 PMC2859614

[27] Wagner AK, Soumerai SB, Zhang F, Ross-Degnan D. Segmented regression analysis of interrupted time series studies in medication use research. J CLIN PHARM THER, 2002;27(4):299–309. 12174032 10.1046/j.1365-2710.2002.00430.x

[28] Lal S, Ndyomugenyi R, Alexander ND, Lagarde M, Paintain L, Magnussen P et al Health Facility Utilisation Changes during the Introduction of Community Case Management of Malaria in South Western Uganda: An Interrupted Time Series Approach. PLoS One. 2015; 10(9):e0137448 doi: 10.1371/journal.pone.0137448 26356099 10.1371/journal.pone.0137448 PMC4565684

[29] Hildreth C, LU John Y.. Demand relations with autocorrelated disturbances. Michigan Stata University; 1960.

[30] Koenker R. A note on studentizing a test for heteroscedasticity. J ECONOMETRICS, 1981;17(1):107–12. doi: 10.1016/0304-4076(81)90062-2

[31] Xu K, Evans DB, Carrin G, Aguilar-Rivera AM, Musgrove P, Evans T. Protecting households from catastrophic health spending. Health Aff (Millwood), 2007;26(4):972–83. doi: 10.1377/hlthaff.26.4.972 17630440 10.1377/hlthaff.26.4.972

[32] Liu Y, Rao K, Wu J, Gakidou E. Health System Reform in China 7 China’s health system performance. LANCET, 2008;372(9653):1914–23. 18930536 10.1016/S0140-6736(08)61362-8

[33] Yip CM, Hsiao WC, Chen W, Hu S, Ma J, Maynard A. Early appraisal of China’s huge and complex health-care reforms. LANCET, 2012;379(9818):833–42. doi: 10.1016/S0140-6736(11)61880-1 22386036 10.1016/S0140-6736(11)61880-1

[34] Ministry of Human Resources and Social Security, China. Statistical Bulletin of China Labor and Social Security 1998. [Internet].1998 http://www.mohrss.gov.cn/SYrlzyhshbzb/zwgk/szrs/tjgb/200602/t20060207_69891.html.

[35] Meng Q, Fang H, Liu X, Yuan B, Xu J. Consolidating the social health insurance schemes in China: towards an equitable and efficient health system. LANCET, 2015;386(10002):1484–92. doi: 10.1016/S0140-6736(15)00342-6 26466052 10.1016/S0140-6736(15)00342-6

[36] National Bureau of Statistics, China. Revenue and Expenses of Urban Basic Medical Care Insurance by Region (2014). [Internet].2015 http://www.stats.gov.cn/tjsj/ndsj/2015/indexeh.htm.

[37] Ministry of Human Resources and Social Security, China. Proposals on further advancing the reform of payment methods for medical insurance. [Internet].2011 http://www.gov.cn/gongbao/content/2011/content_2004738.htm.

[38] National Health And Family Planning Commission, China. Proposals on promoting the reform of payment methods for New Rural Cooperative Medical Scheme. [Internet]. 2012 http://www.mof.gov.cn/zhengwuxinxi/zhengcefabu/201205/t20120516_651635.htm.

[39] Huang Y. The Sick Man of Asia: China’s Health Crisis. Foreign Affairs, 2011(6):119–36. Stable URL: http://www.jstor.org/stable/23039634

[40] Yip CM, Hsiao WC, Chen W, Hu S, Ma J, Maynard A. Early appraisal of China’s huge and complex health-care reforms. LANCET, 2012;379(9818):833–42. doi: 10.1016/S0140-6736(11)61880-1 22386036 10.1016/S0140-6736(11)61880-1

[41] You X, Kobayashi Y. The new cooperative medical scheme in China. HEALTH POLICY, 2009;91(1):1–9. doi: 10.1016/j.healthpol.2008.11.012 19121873 10.1016/j.healthpol.2008.11.012

[42] Babiarz KS, Miller G, Yi H, Zhang L, Rozelle S. New evidence on the impact of China’s New Rural Cooperative Medical Scheme and its implications for rural primary healthcare: multivariate difference-in-difference analysis. BMJ, 2010;341:c5617 doi: 10.1136/bmj.c5617 20966008 10.1136/bmj.c5617 PMC6173169

[43] Zou J, Yang W, Cook DM, Yuan Z, Zhang L, Wang X. New cooperative medical financing policy and hospitalization in rural China: multi-stage cross-sectional surveys. INT HEALTH, 2015;8(1). doi: 10.1093/inthealth/ihv029 26045482 10.1093/inthealth/ihv029

[44] Wagstaff A, Lindelow M, Jun G, Ling X, Juncheng Q. Extending Health Insurance To The Rural Population: An Impact Evaluation Of Chinas New Cooperative Medical Scheme. J HEALTH ECON, 2009;28(1):1–19. doi: 10.1016/j.jhealeco.2008.10.007 19058865 10.1016/j.jhealeco.2008.10.007

[45] World Health Organization. World health statistics. [Internet]. 2017 http://www.who.int/gho/publications/world_health_statistics/en/.

[46] Davis C, Rhodes DJ. The impact of DRGs on the cost and quality of health care in the United States. HEALTH POLICY, 1988;9(2):117–31. 10312505 10.1016/0168-8510(88)90029-2

[47] Yip W, Eggleston K. Addressing government and market failures with payment incentives: Hospital reimbursement reform in Hainan, China. SOC SCI MED, 2004;58(2):267–77. doi: 10.1016/S0277-9536(03)00010-8 14604613 10.1016/s0277-9536(03)00010-8

[48] Li Y, Wu Q, Xu L, et al Factors affecting catastrophic health expenditure and impoverishment from medical expenses in China: policy implications of universal health insurance. B WORLD HEALTH ORGAN, 2012;90(9):664–71. doi: 10.2471/BLT.12.102178 22984311 10.2471/BLT.12.102178 PMC3442391

[49] Cui B, Wang L, Xiong X, Li J. HS3—Had the Individual Medical Burden of Basic Health Insurance Participants Really Been Alleviated in 2009–2012? VALUE HEALTH, 2014;17(7):A721 doi: 10.1016/j.jval.2014.08.022 27202558 10.1016/j.jval.2014.08.022

[50] Ma J, Xu J, Zhang Z, Jing W. New cooperative medical scheme decreased financial burden but expanded the gap of income-related inequity: evidence from three provinces in rural China. International Journal for Equity in Health, 2016;15(1):1–11. doi: 10.1186/s12939-016-0361-5 27142618 10.1186/s12939-016-0361-5 PMC4855492

[51] Liu X, Tang S, Yu B, et al Can rural health insurance improve equity in health care utilization? a comparison between China and Vietnam. International Journal for Equity in Health, 2012;11(1):1–9. doi: 10.1186/1475-9276-11-10 22376290 10.1186/1475-9276-11-10 PMC3334712

[52] Yang X, Gao J, Zhou Z, et al Assessing the Effects of the New Cooperative Medical Scheme on Alleviating the Health Payment-Induced Poverty in Shaanxi Province, China. PLOS ONE, 2016;11(7). doi: 10.1371/journal.pone.0157918 27380417 10.1371/journal.pone.0157918 PMC4933374

[53] Liu K, Wu Q, Liu J. Examining the association between social health insurance participation and patients’ out-of-pocket payments in China: The role of institutional arrangement. SOC SCI MED, 2014;113(1982):95–103. doi: 10.1016/j.socscimed.2014.05.011 24858927 10.1016/j.socscimed.2014.05.011

[54] Barber SL, Yao L. Health insurance systems in China: A briefing note. World Health Report (2010). Background Paper 37. Geneva, Switzerland: World Health Organization; [Internet]. 2010 http://www.who.int/healthsystems/topics/financing/healthreport/37ChinaB_YFINAL.pdf. Accessed 10–8.

[55] Li C, Yu X, Butler JR, Yiengprugsawan V, Yu M. Moving towards universal health insurance in China: performance, issues and lessons from Thailand. SOC SCI MED, 2011;73(3):359–66. doi: 10.1016/j.socscimed.2011.06.002 21733610 10.1016/j.socscimed.2011.06.002

