# Supplementary material for: Impact of universal medical insurance system on the accessibility of medical service supply and affordability of patients in China
Source: PLoS One. 2018 Mar 7;13(3):e0193273. doi: 10.1371/journal.pone.0193273 (PMC5841764; doi:10.1371/journal.pone.0193273)
Supplement: S1 File — (DOC) [file pone.0193273.s001.doc]

**Table1. Estimated coefficients of segmented regression model for revenue and expenditure of UMIS funding before and after 2008 in China.**

| **Coefficient** | **Revenue of UMIS adjusted by CPI (Billion RMB)** | **Expenditure of UMIS adjusted by CPI (Billion RMB)** |
| --- | --- | --- |
| **β_0_(Intercept)** | 18.055 | 13.633 |
| **β_1_(Slope pre- UMIS)** | 33.682*** | 24.490*** |
| **β_2_(Level change after UMIS)** | -37.034 | -46.912** |
| **β_3_ (Slope change after UMIS)** | 69.442*** | 71.424*** |
| **β_1_+β_3_(Slope post- UMIS)** | 103.124 | 95.914 |

Significant codes (P value of two-sided test) : <0.01‘***’, <0.05‘**’, <0.1‘*’.

**Table2. Estimated coefficients of segmented regression model for fatality rates of eight diseases in the general hospitals before and after the UMIS.**

| **Coefficient** | **Fatality rate of Acute Myocardial Infarction (%)** | **Fatality rate of Heart Failure (%)** | **Fatality rate of Pneumonia (%)** | **Fatality rate of Leukemia (%)** | **Fatality rate of Rheumatic heart disease (%)** | **Fatality rate of Cerebro**  **vascular disease (%)** | **Fatality rate of Diabetes mellitus (%)** | **Fatality rate of Tuberculosis (%)** |
| --- | --- | --- | --- | --- | --- | --- | --- | --- |
| **β_0_(Intercept)** | 10.338  *** | 10.107  *** | 1.006  *** | 8.314  *** | 3.361  *** | 5.350  *** | 1.380  *** | 1.391  *** |
| **β_1_(Slope pre- UMIS)** | -0.008 | -0.804  *** | -0.051  ** | -0.752  ** | -0.202  ** | -0.376  *** | -0.092  * | -0.059  * |
| **β_2_(Level change after UMIS)** | 0.649 | 1.597  *** | 0.071 | 0.527 | 0.353 | 0.115 | 0.001 | -0.018 |
| **β_3_(Slope change after UMIS)** | -0.835  *** | 0.213 | 0.019 | 0.571 | -0.001 | 0.048 | 0.007 | -0.036 |
| **β_1_+β_3_(Slope post- UMIS)** | -0.843 | -0.591 | -0.032 | -0.181 | -0.203 | -0.328 | -0.085 | -0.095 |

Significant codes (P value of two-sided test) : <0.01‘***’, <0.05‘**’, <0.1‘*’.
