# Supplementary material for: Impact of universal medical insurance system on the accessibility of medical service supply and affordability of patients in China
Source: PLoS One. 2018 Mar 7;13(3):e0193273. doi: 10.1371/journal.pone.0193273 (PMC5841764; doi:10.1371/journal.pone.0193273)
Supplement: S2 File — (DOCX) [file pone.0193273.s002.docx]

S2 files. Original data in the study.

**Table1. Annual revenue on UMIS funding in China from 2002 to 2014.**

| **Year** | **Revenue of UEBMI adjusted by CPI (Billion RMB)** | **Revenue of URBMI adjusted by CPI (Billion RMB)** | **Revenue of NRCMS adjusted by CPI (Billion RMB)** | **Revenue of UMIS adjusted by CPI (Billion RMB)** |
| --- | --- | --- | --- | --- |
| **2002** | 60.78 | N/A | N/A | 60.78 |
| **2003** | 87.94 | N/A | N/A | 87.94 |
| **2004** | 108.47 | N/A | 3.83 | 112.30 |
| **2005** | 131.29 | N/A | 7.04 | 138.33 |
| **2006** | 160.81 | N/A | 19.66 | 180.47 |
| **2007** | 194.47 | 3.78 | 37.59 | 235.83 |
| **2008** | 239.31 | 12.85 | 65.07 | 317.22 |
| **2009** | 285.66 | 21.01 | 78.88 | 385.55 |
| **2010** | 319.80 | 28.58 | 105.78 | 454.16 |
| **2011** | 379.32 | 45.58 | 157.06 | 581.97 |
| **2012** | 453.22 | 65.55 | 185.77 | 704.53 |
| **2013** | 514.58 | 86.47 | 216.60 | 817.65 |
| **2014** | 574.24 | 117.83 | 216.13 | 908.19 |

Note: annual revenue amount of UEBMI and URBMI between 2002 and 2014 extracted from the China Statistical Yearbook 2003 to 2015. Annual revenue amount of NRCMS between 2002 and 2014 extracted from the China Health Statistics Yearbook 2003 to 2015. CPI from 2002 to 2014 is 1, 1.01, 1.05, 1.07, 1.09, 1.14, 1.21, 1.20, 1.24, 1.30, 1.34, 1.37, 1.40 and 1.42.

**Table2. Annual expenditure on UMIS funding in China from 2002 to 2014.**

| **Year** | **Expenditure of UEBMI adjusted by CPI (Billion RMB)** | **Expenditure of URBMI adjusted by CPI (Billion RMB)** | **Expenditure of NRCMS adjusted by CPI (Billion RMB)** | **Expenditure of UMIS adjusted by CPI (Billion RMB)** |
| --- | --- | --- | --- | --- |
| **2002** | 40.94 | N/A | N/A | 40.94 |
| **2003** | 64.61 | N/A | N/A | 64.61 |
| **2004** | 82.00 | N/A | 2.51 | 84.51 |
| **2005** | 100.78 | N/A | 5.77 | 106.55 |
| **2006** | 117.52 | N/A | 14.34 | 131.86 |
| **2007** | 136.28 | 0.89 | 30.44 | 167.61 |
| **2008** | 167.50 | 5.30 | 54.90 | 227.70 |
| **2009** | 219.66 | 13.97 | 77.08 | 310.72 |
| **2010** | 264.51 | 21.55 | 96.03 | 382.09 |
| **2011** | 308.23 | 31.69 | 131.19 | 471.11 |
| **2012** | 363.99 | 50.48 | 180.03 | 594.50 |
| **2013** | 424.83 | 70.76 | 211.91 | 707.50 |
| **2014** | 478.41 | 102.66 | 206.49 | 787.57 |

Note: annual expenditure amount of UEBMI and URBMI between 2002 and 2014 extracted from the China Statistical Yearbook 2003 to 2015. Annual expenditure amount of NRCMS between 2002 and 2014 extracted from the China Health Statistics Yearbook 2003 to 2015. CPI from 2002 to 2014 is 1, 1.01, 1.05, 1.07, 1.09, 1.14, 1.21, 1.20, 1.24, 1.30, 1.34, 1.37, 1.40 and 1.42.

**Table3. Annual number of medical service supply per 1,000 individuals in China from 2002 to 2014**

| **Year** | **Number of licensed physicians and assistants per 1,000 individuals** | **Number of urban licensed physicians and assistants per 1,000 urban individuals** | **Number of rural licensed physicians and assistants per 1,000 rural individuals** | **Number of licensed nurses per 1,000 individuals** | **Number of urban licensed nurses per 1,000 urban individuals** | **Number of rural licensed nurses per 1,000 rural individuals** | **Number of beds per 1,000 individuals** | **Number of beds in township hospitals per 1,000 rural individuals** |
| --- | --- | --- | --- | --- | --- | --- | --- | --- |
| **2002** | 1.09 | N/A | N/A | 0.85 | N/A | N/A | 2.28 | 0.74 |
| **2003** | 1.09 | 2.13 | 1.04 | 0.86 | 1.59 | 0.5 | 2.31 | 0.76 |
| **2004** | 1.10 | 2.18 | 1.04 | 0.88 | 1.63 | 0.5 | 2.37 | 0.76 |
| **2005** | 1.11 | 2.46 | 1.26 | 0.90 | 2.1 | 0.65 | 2.42 | 0.78 |
| **2006** | 1.15 | 2.56 | 1.26 | 0.94 | 2.22 | 0.66 | 2.53 | 0.80 |
| **2007** | 1.18 | 2.61 | 1.23 | 1.02 | 2.42 | 0.7 | 2.67 | 0.85 |
| **2008** | 1.23 | 2.68 | 1.26 | 1.09 | 2.54 | 0.76 | 2.90 | 0.96 |
| **2009** | 1.31 | 2.83 | 1.31 | 1.21 | 2.82 | 0.81 | 3.16 | 1.05 |
| **2010** | 1.37 | 2.97 | 1.32 | 1.34 | 3.09 | 0.89 | 3.42 | 1.12 |
| **2011** | 1.39 | 3 | 1.33 | 1.47 | 3.29 | 0.98 | 3.67 | 1.16 |
| **2012** | 1.48 | 3.19 | 1.4 | 1.63 | 3.65 | 1.09 | 4.05 | 1.24 |
| **2013** | 1.55 | 3.39 | 1.48 | 1.80 | 4 | 1.22 | 4.36 | 1.30 |
| **2014** | 1.61 | 3.54 | 1.51 | 1.94 | 4.3 | 1.31 | 4.64 | 1.34 |

Note: annual numbers of licensed physicians and assistant physicians, nurses, hospital beds per 1,000 individuals between 2002 and 2014 extracted from the China Health Statistics Yearbook 2003 to 2015.

**Table4. Annual average number of outpatient visits in different level hospitals in China from 2002 to 2014.**

| **Year** | **Number of outpatient visits per hospital** | **Number of outpatient visits per general hospital** | **Number of outpatient visits per community center** | **Number of outpatient visits per township hospital** | **Number of outpatient visits per village clinic** |
| --- | --- | --- | --- | --- | --- |
| **2002** | 69662.61 | 75828.55 | 51847.86 | 15777.64 | . |
| **2003** | 68269.20 | 73858.92 | 50523.04 | 15613.27 | . |
| **2004** | 70925.18 | 77104.33 | 40918.35 | 16349.74 | 2237.14 |
| **2005** | 74134.28 | 81478.16 | 42970.47 | 16604.33 | 2116.08 |
| **2006** | 76432.14 | 84720.47 | 39891.57 | 17533.03 | 2213.64 |
| **2007** | 82495.26 | 92175.20 | 40229.26 | 19022.94 | 2259.11 |
| **2008** | 90385.03 | 102220.01 | 42733.65 | 21156.62 | 2232.62 |
| **2009** | 94718.78 | 107423.84 | 50000.46 | 22783.84 | 2452.24 |
| **2010** | 97506.13 | 110414.58 | 50326.54 | 23105.00 | 2555.46 |
| **2011** | 102772.52 | 116839.81 | 52092.55 | 23233.646 | 2763.72 |
| **2012** | 109694.26 | 124727.38 | 55579.45 | 26082.37 | 2907.07 |
| **2013** | 110962.68 | 126881.40 | 59835.75 | 27208.63 | 3079.47 |
| **2014** | 114929.23 | 132046.10 | 61851.19 | 27875.43 | 3062.33 |

Note: annual numbers of different hospitals and total numbers of outpatient visits in different-level hospitals between 2002 and 2014 extracted from the China Health Statistics Yearbook 2003 to 2015.

**Table5. Annual average number of inpatient visits in different level hospitals in China from 2002 to 2014.**

| **Year** | **Number of inpatient visits per hospital** | **Number of inpatient visits per general hospital** | **Number of inpatient visits per community center** | **Number of inpatient visits per township hospital** |
| --- | --- | --- | --- | --- |
| **2002** | 2239.93 | 2593.00 | 153.31 | 361.22 |
| **2003** | 2341.21 | 2682.08 | 136.58 | 363.06 |
| **2004** | 2540.83 | 2945.57 | 134.72 | 384.25 |
| **2005** | 2731.15 | 3198.83 | 192.63 | 396.49 |
| **2006** | 2890.05 | 3414.65 | 210.06 | 459.30 |
| **2007** | 3267.78 | 3880.89 | 235.19 | 667.61 |
| **2008** | 3750.01 | 4475.72 | 255.89 | 847.68 |
| **2009** | 4183.15 | 5023.20 | 314.88 | 989.66 |
| **2010** | 4552.91 | 5486.05 | 315.89 | 959.50 |
| **2011** | 4893.19 | 5884.49 | 314.65 | 924.73 |
| **2012** | 5493.07 | 6600.67 | 328.35 | 1053.32 |
| **2013** | 5668.96 | 6828.21 | 344.09 | 1063.66 |
| **2014** | 5945.53 | 7167.84 | 343.82 | 1011.49 |

Note: annual numbers of different hospitals and total numbers of inpatient visits in different-level hospitals between 2002 and 2014 extracted from the China Health Statistics Yearbook 2003 to 2015.

**Table6. Annual average fatality rates of inpatients in different level hospitals in China from 2002 to 2014.**

| **Year** | **Inpatient fatality rate in all hospitals（%）** | **Inpatient fatality rate in generic hospitals（%）** | **Inpatient fatality rate in community centers（%）** | **Inpatient fatality rate in township hospitals（%）** |
| --- | --- | --- | --- | --- |
| **2002** | 1.11 | 1.17 | 2.41 | 0.17 |
| **2003** | 1.15 | 1.23 | 3.40 | 0.18 |
| **2004** | 1.11 | 1.18 | 3.22 | 0.17 |
| **2005** | 1.08 | 1.14 | 2.34 | 0.16 |
| **2006** | 1.03 | 1.10 | 1.76 | 0.13 |
| **2007** | 1.00 | 1.00 | 1.10 | 0.10 |
| **2008** | 0.90 | 0.90 | 1.00 | 0.10 |
| **2009** | 0.80 | 0.80 | 0.70 | 0.10 |
| **2010** | 0.70 | 0.80 | 0.50 | 0.10 |
| **2011** | 0.70 | 0.70 | 0.50 | 0.10 |
| **2012** | 0.42 | 0.44 | 0.41 | 0.04 |
| **2013** | 0.45 | 0.47 | 0.39 | 0.04 |
| **2014** | 0.50 | 0.50 | 0.40 | 0.00 |

Note: annual fatality rates of inpatients in different-level hospitals between 2002 and 2014 extracted from the China Health Statistics Yearbook 2003 to 2015.

**Table7. Annual average fatality rates of eight diseases in the general hospitals in China from 2002 to 2014.**

| **Year** | **Fatality rate of acute myocardial infarction (%)** | **Fatality rate of heart failure (%)** | **Fatality rate of pneumonia (%)** | **Fatality rate of leukemia (%)** | **Fatality rate of rheumatic heart disease (%)** | **Fatality rate**  **of cerebrovascular disease (%)** | **Fatality rate of diabetes mellitus (%)** | **Fatality rate of tuberculosis (%)** |
| --- | --- | --- | --- | --- | --- | --- | --- | --- |
| **2002** | 9.96 | 9.22 | 1.03 | 9.12 | 3.44 | 5.18 | 1.10 | 1.48 |
| **2003** | 10.10 | 8.04 | 0.84 | 5.87 | 2.69 | 4.12 | 1.34 | 1.23 |
| **2004** | 10.60 | 8.30 | 0.80 | 4.60 | 2.60 | 4.30 | 1.20 | 1.10 |
| **2005** | 10.00 | 6.60 | 0.80 | 5.00 | 2.40 | 3.90 | 1.00 | 1.00 |
| **2006** | 12.80 | 7.10 | 0.80 | 5.50 | 2.80 | 4.00 | 1.00 | 1.20 |
| **2007** | 8.40 | 4.50 | 0.70 | 4.00 | 2.00 | 2.70 | 0.70 | 1.10 |
| **2008** | 10.10 | 6.00 | 0.70 | 4.40 | 2.40 | 3.10 | 0.80 | 0.90 |
| **2009** | 9.30 | 5.90 | 0.80 | 3.90 | 2.00 | 2.50 | 0.70 | 0.90 |
| **2010** | 8.20 | 5.00 | 0.70 | 3.70 | 1.90 | 2.10 | 0.50 | 0.70 |
| **2011** | 7.50 | 4.80 | 0.60 | 3.50 | 1.70 | 1.80 | 0.40 | 0.60 |
| **2012** | 7.41 | 4.43 | 0.57 | 3.28 | 1.47 | 1.50 | 0.38 | 0.55 |
| **2013** | 5.27 | 2.69 | 0.51 | 3.00 | 1.21 | 1.12 | 0.31 | 0.45 |
| **2014** | 5.18 | 2.82 | 0.64 | 3.45 | 1.18 | 1.16 | 0.31 | 0.36 |

Note: annual fatality rates of eight diseases in the general hospitals between 2002 and 2014 extracted from the China Health Statistics Yearbook 2003 to 2015.

**Table8. Annual average inpatient medical expenses and reimbursement rates of UMIS beneficiaries in China from 2008 to 2014.**

| **Year** | **UEBMI beneficiaries** | | **URBMI beneficiaries** | | **NRCMS beneficiaries** | |
| --- | --- | --- | --- | --- | --- | --- |
|  | **Average inpatient expense (RMB)** | **Reimbursement rate (%)** | **Average inpatient expense (RMB)** | **Reimbursement rate (%)** | **Average inpatient expense (RMB)** | **Reimbursement rate (%)** |
| **2008** | 6877 | 66.21 | 4510 | 38.01 | 2800 | 38 |
| **2009** | 7764 | 69.58 | 7252 | 54.20 | 2981 | 41 |
| **2010** | 8903 | 70.62 | 5599 | 50.98 | 3371 | 43 |
| **2011** | 8849 | 71.40 | 5941 | 52.48 | 3912. | 48 |
| **2012** | 9732 | 69.63 | 5911 | 52.05 | 4145 | 55 |
| **2013** | 10327 | 71.90 | 6807 | 54.43 | 4398 | 57 |
| **2014** | 10838 | 72.50 | 7508 | 54.74 | 4702 | 54 |

Note: annual average medical expenses and reimbursement rates of inpatients covered by UEBMI, URBMI and NRCMS from 2008 to 2014 were obtained from the national sample survey on medical service utilization of basic medical insurance participants of the China Health Insurance Research Association and New Rural Cooperative Medical Research Center of China.

**Table9. Annual average disposable incomes of populations with three levels in China from 2008 to 2014.**

| **Year** | **Average income of 20% lowest income population (RMB)** | | **Average income of 20% middle income population (RMB)** | | **Average income of 20% highest income population (RMB)** | |
| --- | --- | --- | --- | --- | --- | --- |
|  | urban | rural | urban | rural | urban | rural |
| **2008** | 6075 | 1500 | 13984 | 4203 | 34668 | 11290 |
| **2009** | 6725 | 1549 | 15400 | 4502 | 37434 | 12319 |
| **2010** | 7605 | 1870 | 17224 | 5222 | 41158 | 14050 |
| **2011** | 8789 | 2001 | 19545 | 6208 | 47021 | 16783 |
| **2012** | 10354 | 2316 | 22419 | 7041 | 51456 | 19009 |
| **2013** | 11434 | 2583 | 24518 | 7942 | 56389 | 21273 |
| **2014** | 11219 | 2768 | 26651 | 9504 | 61615 | 23947 |

Note: three levels of disposable incomes of residents were obtained between 2008 and 2014 from the China Statistical Yearbook 2009 to 2015.
